# Discovery of the first PD-1 ligand encoded by a pathogen

**DOI:** 10.3389/fimmu.2022.1007334

**Published:** 2022-09-13

**Authors:** Pablo Martínez-Vicente, Francesc Poblador, Judith Leitner, Domènec Farré, Peter Steinberger, Pablo Engel, Ana Angulo

**Affiliations:** ^1^Unitat d’Immunologia, Departament de Biomedicina, Facultat de Medicina i Ciències de la Salut, Universitat de Barcelona (UB), Barcelona, Spain; ^2^Division of Immune Receptor and T-Cell Activation, Institute of Immunology, Center for Pathophysiology, Infectiology and Immunology, Medical University of Vienna, Vienna, Austria; ^3^Institut d’Investigacions Biomèdiques August Pi i Sunyer (IDIBAPS), Barcelona, Spain

**Keywords:** PD-1 inhibitory receptor, viral PD-1 ligand homolog, γ-herpesvirus, viral immunosuppressive strategy, host-pathogen interaction

## Abstract

Large double-stranded DNA viruses deploy multiple strategies to subvert host immune defenses. Some of these tactics are mediated by viral gene products acquired by horizontal gene transfer from the corresponding hosts and shaped throughout evolution. The programmed death-1 (PD-1) receptor and its ligands, PD-L1 and PD-L2, play a pivotal role attenuating T-cell responses and regulating immune tolerance. In this study, we report the first functional PD-L1 homolog gene (De2) found in a pathogen. De2, captured by a γ-herpesvirus from its host during co-evolution around 50 million years ago, encodes a cell-surface glycoprotein that interacts with high affinity and stability with host PD-1. We also find that mutations evolved by the viral protein result in a significant loss of its ability to interact in *cis* with CD80, an interaction that for PD-L1:CD80 has been reported to block PD-1 inhibitory pathways. Furthermore, we demonstrate that the viral protein strongly inhibits T-cell signaling. Our observations suggest that PD-L1 homologs may enable viruses to evade T cell responses, favor their replication, and prevent excessive tissue damage. Altogether, our findings reveal a novel viral immunosuppressive strategy and highlight the importance of the modulation of the PD-1/PD-L1 axis during viral infections.

## Introduction

Viruses have evolved a wide range of tactics to counteract host immunity. In particular, large DNA viruses, such as herpesviruses, encode a rich arsenal of gene products that efficiently blunt immune effector responses in order to establish successful lifelong persistent infections (1, 2). Some of these strategies are mediated by viral genes originally acquired from their hosts *via* horizontal gene transfer (3, 4). Classical examples of virally encoded homologs of cellular immune proteins associated with immune responses include homologs of MHC class I, Fc receptors, cytokines and their receptors (4, 5).

Programmed death 1 (PD-1, CD279) is a receptor located at the surface of activated T and B cells. It inhibits activated T cells, primarily in peripheral tissues, representing an important regulator of immune tolerance and T cell exhaustion (6). The ligands of PD-1, PD-L1 (CD274) and PD-L2 (CD273), are type I glycoproteins that belong to the family of B7 co-stimulatory molecules. PD-L1 is constitutively expressed on hematopoietic and non-hematopoietic cells, including antigen-presenting cells (APCs), and can be further regulated by extracellular stimuli. PD-1 and PD-L1 contain canonical immunoglobulin (Ig)-like extracellular domains responsible for their interaction (7). *Via* an immunoreceptor tyrosine-based inhibitory motif and an immunoreceptor tyrosine-based switch motif in its intracellular domain, PD-1 exerts its functions (6). Activation of the PD-1/PD-L1 pathway is frequently employed by tumors to escape from immune eradication, and consequently this pathway has become a crucial target in the treatment of cancer (8).

Here, we hypothesized that viruses have captured during host-coevolution co-inhibitory molecules, such as PD-1 ligands, to use them as part of their immune evasion toolbox. We describe the presence of a functional PD-L1 gene in a mammal γ-herpesvirus, capable to efficiently engage host PD-1 and potently suppress T cell activation. Altogether, this work reveals a novel viral mechanism to subvert immune surveillance.

## Material and methods

### Identification of De2 as a CD274 (PD-L1) homolog and sequence analysis

To search for viral genes homologous to *PD-L1* and *PD-L2*, NCBI’s blastn and DELTA-BLAST were used for mRNA and protein sequences, respectively (http://blast.ncbi.nlm.nih.gov). Cetacean protein sequences of PD-L1 were downloaded from GenBank (9). Open reading frames (ORF) of *Tursiops truncatus gammaherpervirus 1* (TTGHV1) were extracted from RefSeq file NC_035117.1. De2 protein sequence was obtained from RefSeq file YP_009388506.1. Nucleotide and protein sequence alignments were calculated using MAFFT version 7.475 (10).

### Protein domain, motif prediction, and structure modeling

Ig domains were identified by using annotations in CDD (11). Signal peptides and transmembrane regions were determined by using SignalP 4.1 [https://services.healthtech.dtu.dk/service.php?SignalP-4.1 ] and TMHMM 2.0 [https://services.healthtech.dtu.dk/service.php?TMHMM-2.0], respectively. Protein N-glycosylation sites were predicted by using NetNGlyc 1.0 [https://services.healthtech.dtu.dk/service.php?NetNGlyc-1.0 ]. To calculate the percentages of amino acid identities of each region, pair alignment of De2 and *Tursiops truncatus* PD-L1 protein sequences was obtained. Structure modeling of De2 and PD-L1 proteins was performed using SWISS-MODEL with the template 3sbw of the human PD-L1 structure (PDB: 3SBW) (12).

### Bayesian phylogenetic analysis

The PD-L1 capture moment, origin of De2, was estimated by applying BEAST (v1.10.4) with the Bayesian random local clocks method (13), using a cDNA alignment previously obtained with MAFFT (10). BEAST was run 12 times (estimating 8000 trees for each run) and a consensus tree was generated by combining the results of the 12 runs using the LogCombiner (v1.10.4) program of the BEAST software package. The HKY85 model (14) was used for nucleotide substitution with a discrete gamma distribution (5 categories) to model evolutionary rate differences among sites. A maximum likelihood phylogenetic tree obtained from the cDNA alignment applying RAxML with 500 bootstrap replicates was used as a starting tree model of the BEAST estimation (15). Priors for the time to the most recent common ancestor of Bovidae (19.1 million years ago [mya], standard deviation [SD] 0.7 mya), Bovini (10.7 mya, SD 0.8 mya), Camelidae (14.5 mya, SD 3.0 mya), Cetartiodactyla (67.7 mya, SD 2.5 mya), Delphinidae (6.6 mya, SD 0.7 mya), Delphinoidea (15.0 mya, SD 1.5 mya), Neoceti (36.4 mya, SD 0.3 mya), Odontoceti (32.6 mya, SD 1.6 mya), and Pecora (26.4 mya, SD 1.5 mya) were set according to the bibliography (16).

### Expression plasmids

The following plasmids were constructed as indicated in detail in Supplementary information: HA-PD-L1, HA-CD80, and HA-De2, expressing the full-length proteins (from *Tursiops truncatus* or TTGHV1*)* without their corresponding signal peptides and with the HA epitope at their N-terminal ends, and HA-chiPD-1, a chimera containing the N-terminal HA-tagged ectodomain of the dolphin PD-1 (*Lagenorhynchus obliquidens* sp.) fused to the transmembrane domain and cytoplasmic tail of human PD-1, were cloned in the pDisplay vector (Invitrogen); host PD-1-Fc, De2-Fc and human PD-L1-Fc fusion proteins, expressing the single (for PD-1) or two (for PD-L1 and De2) Ig-like domains of these molecules (with the CD33 leader peptide replacing their own signal peptide) fused to the Fc region of human IgG_1_, in the pCI-neo-Fc vector (17); De2-GFP and PD-L1-GFP, expressing De2 or PD-L1 fused to the GFP gene at the C-terminal end, De2/PD-L1-GFP chimera, a PD-L1-GFP molecule in which the first Ig-like domain was exchanged by the first Ig-like domain of De2, and PD-L1 VD-GFP, a mutant PD-L1-GFP with residues L^74^ and S^79^ replaced by the corresponding ones in De2, V^74^ and D^79^, in the pEGFP-N3 vector (BD Biosciences Clontech). Detailed descriptions of the expression constructs for the T cell stimulator cells, De2 and PD-L1 HA-tagged into the retroviral vector pCJK2, and for the reporter cells, chiPD-1 HA-tagged into the lentivirus vector pHR, are also given in Supplementary information. The primers used for the generation of all plasmids are included in **Supplementary Table 1**.

### Cell culture

The cell lines COS-7 (green monkey fibroblast) and HEK-293T (human embryonic kidney) were obtained from the American Type Culture Collection and cultured in Dulbecco’s modified Eagle’s medium supplemented with 2 mM glutamine, 1 mM sodium pyruvate, 50 U of penicillin per ml, 50 g of streptomycin per ml, and 10% fetal bovine serum (Sigma). Bw5147 (short designation Bw) and Jurkat cells were cultured in RPMI-1640 medium supplemented with 2 mM glutamine, 100 U of penicillin per ml, 100 μg/ml streptomycin and 10% of fetal calf serum (Sigma). The Jurkat NF-kB::eGFP monoreporter cell line was described previously (18). T cell stimulator cells used in this study are based on the Bw cell line and express membrane-bound single chain antibody fragments derived from the CD3 antibodies UCHT1 or OKT3 on their surface (19).

### Antibodies

The mouse anti-HA monoclonal antibody (mAb) and the mouse anti-human IgG (29.5; Fc specific; anti-Fc) were generated in our lab and have been previously described (17). The anti-human IgG (Fc specific) labelled with peroxidase (POD) was from Sigma-Aldrich, the anti-human PD-1 (clone EH12.2H7) from BioLegend, and the anti-mouse IgG-PE and IgG-APC from Jackson ImmunoResearch. The rabbit anti-HA (clone C29F4) and the anti-β-actin (clone C4) mAbs, and the anti-rabbit IgG-POD, and the anti-mouse IgG-HRP, were from Cell Signaling, MP Biomedicals, Promega, and Sigma-Aldrich, respectively. The streptavidin-AF555 and the anti-rabbit IgG (H+L)-biotinylated were from Life Technologies and Jackson Immunoresearch, respectively. The anti-human CD14-APC (clone M5E2) mAb employed to stain surface expression of anti-CD3 single chain fragments, and the anti-mouse CD45-APC (clone 104) mAb used to exclude the TCS in the reporter assays, were obtained from BioLegend.

### Transfections and generation of Ig fusion proteins

COS-7 cells were transiently transfected or co-transfected with 5-10 µg of the indicated plasmids using the Amaxa Cell Line Nucleofector Kit R (Lonza) according to the manufacturer’s protocol. For co-transfection assays with HA-CD80 or empty pDisplay plasmids and GFP-expressing plasmids, 5 µg and 7.5 µg of the corresponding plasmids were used, respectively. For the production of Fc fusion proteins, HEK-293T cells were transiently transfected with 0.2 µg/cm^2^ of the indicated plasmid mixed with 6 µL/µg DNA of polyethylenimine (1mg/mL, Sigma-Aldrich) in 0.1 mL/cm^2^ of OPTIMEM medium (Gibco) for four hours. Then HEK cell cultures were washed, fresh medium added, and 6 days later Fc fusion proteins were collected from supernatants. Supernatants were further clarified by centrifugation and concentrated 30-fold using the Amicon Ultra-15 Centrifugal Filter Unit with an Ultracel-30 membrane (Millipore). Quantification of Fc fusion proteins was performed by sandwich ELISA using anti-Fc mAb and anti-human IgG-POD (Fc specific). For SPR analyses, Fc fusion proteins were purified from cell supernatants using Affi-Gel protein-A MAPS II kit (Bio-Rad).

### Surface plasmon resonance (SPR) analyses

SPR analyses were performed at 25°C on a Biacore T200 (GE Healthcare) in acetate buffer pH 6.5 and covalently bound to CM4 chips by amine coupling. Briefly, the chip surface was activated with a 1:1 mixture of 0.1 M NHS (N-hydroxysuccinimide) and 0.1 M EDC (3-(N,N-dimethylamino) propyl-N-ethylcarbodiimide) at a flow rate of 5 μl/min, followed by the injection of ligand (PD-1-Fc fusion protein) or buffer for the active or reference channel, respectively. Ethanolamine solution was injected in both channels in order to block the remaining reactive groups of the surface. De2-Fc and PD-L1-Fc fusion proteins were prepared in PBS with 0.05% Tween (PBS-P) in serial dilutions (0, 12.5, 25, 50, 100, 200 and 400 nM). Assays were run in PBS-P buffer at 30 µL/min and samples injected in reference and active channels for 90s and left dissociate for 240s. The complexes formed were regenerated after each cycle with glycine pH 2 during 30s at 10 μL/min. Blanks were included for double referencing. Kinetic and affinity constants were calculated using Biacore T200 evaluation software 2.0 (GE Healthcare) after reference and blank subtraction, and sensorgrams were fitted according to the 1:1 Langmuir model.

### Generation of reporter and stimulator cell lines

HA-tagged De2 and PD-L1 cloned into the retroviral expression vector pCJK2 were stably expressed in the T cell stimulator cells (20). The chimeric molecule HA-chiPD-1 cloned into the lentiviral expression vector pHR was stably expressed in the Jurkat-NFkB::eGFP monoreporter cell line (21–23).

### Reporter assays

Jurkat-NFkB::eGFP monoreporter cells, expressing or not HA-chiPD-1 chimera (5x10^4^/well) were stimulated with TCS or TCS-CD86, expressing HA-De2, HA-PD-L1, or no co-inhibitory molecule as control (2x10^4^/well), or were left unstimulated. Following 24h of co-culture, reporter activity was measured as previously described in detail (24).

### Flow-cytometry analysis

Flow cytometry was performed using standard procedures (25). Transfected COS-7 cells were stained with the corresponding mAbs, or left unstained (ctrl), and incubated with APC of PE labelled anti-mouse IgG antibodies as indicated. Fc fusion protein staining was performed using different concentrations of Ig fusion proteins followed by incubation with anti-Fc and then anti-mouse IgG-PE. An irrelevant Fc fusion protein was used as a negative control. When indicated, in interaction assays using PD-1-Fc, cells expressing similar levels of GFP signal were gated to assess the extent of PD-1 binding. In reporter assays, the reporter activity (eGFP) was analyzed by FACS and the data is shown as geometric mean fluorescence intensity (gMFI). Flow cytometry analysis were performed using a FACSCalibur (BD Biosciences) and the FlowJo software (Tree star Inc).

### Western blot analyses and N-glycosidase treatments

Samples from cell extracts, previously lysed and quantified by BCA Protein Assay Kit (Thermo Scientific), were treated when indicated with an N-glycosidase F deglycosylation kit (Roche Diagnostics GmbH) following the manufacturer’s instructions. Then, samples were subjected to SDS-PAGE in 10% acrylamide gels and subsequently transferred to nitrocellulose membranes (Protran). Membranes were incubated with rabbit anti-HA followed by anti-rabbit IgG labelled with peroxidase. Anti-β-actin mAb, followed by anti-mouse IgG-HRP, was used as loading control. Blots were developed using a SuperSignal^®^ West Pico Chemiluminescent Substrate (Thermo Scientific) according to the manufacturer’s protocol and the ChemiDoc system from BioRad.

### Immunofluorescence confocal microscopy

For immunofluorescence confocal microscopy assays, cells were cultured on glass coverslips in 24-well tissue culture plates, washed with PBS, and fixed with 4% formaldehyde for 10 min. Subsequently, samples were blocked with 6% fetal bovine serum in PBS, followed by incubation with Streptavidin/Biotin Blocking Kit (Vector Laboratories) to prevent the non-specific binding of biotin, according to the manufacturer’s protocol. Then, samples were incubated with the anti-HA mAb, followed by an anti-rabbit IgG (H+L)-biotinylated and streptavidin-AF555. Nuclei were counterstained with the DAPI reagent (Life Technologies). The samples were mounted in ProLong Gold antifade reagent (Invitrogen). Fluorescence images were obtained using a Leica TCS SP5 laser scanning confocal microscope (Leica Microsystems Heidelberg GmbH, Manheim, Germany) equipped with a DMI6000 inverted microscope, blue diode (405nm), Argon (458/476/488/514nm), diode pumped solid state (561nm) and HeNe (594/633nm) lasers and APO 63x oil (NA 1.4) immersion objective lens. DAPI and Alexa Fluor 561 images were acquired sequentially using 405- and 561 laser lines, AOBS (Acoustic Optical Beam Splitter) as beam splitter and emission detection ranges 415- 480 and 571-625 nm respectively and the confocal pinhole set at 1 Airy units. Images were acquired at 400 Hz at a 1024 x 1024 pixels format.

### Statistical analyses

Analyses were performed with GraphPad Prism software (v.7.03) and Microsoft Excel (2010). Results are given as means ± standard deviations (SD) or standard errors of the means (SEM), and statistical significances were determined with the Student’s t-test (two-tailed). Where indicated, data was analyzed by one-way ANOVA followed by Dunnett’s comparison. P-values less than or equal to 0.05 (*), 0.01 (**), 0.001 (***), and 0.0001 (****) were considered statistically significant.

## Results

### De2, a PD-L1 homolog in a γ-herpesvirus

**Figure 1:**
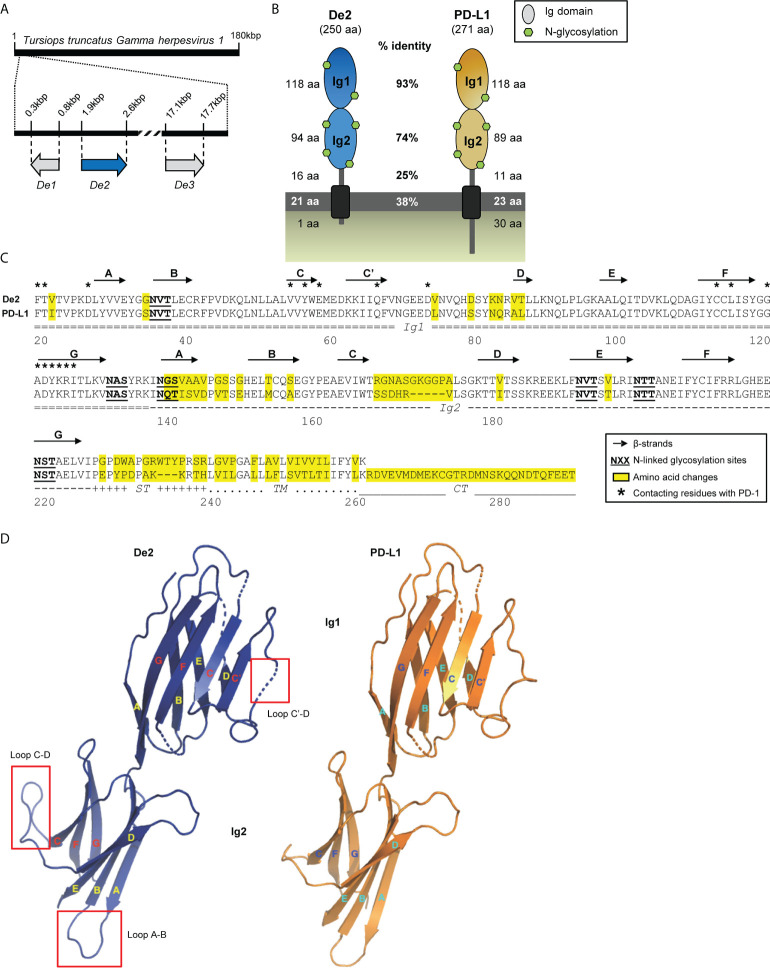
De2, a novel homolog of PD-L1 encoded by TTGHV1. **(A)** Localization of *De2* and surrounding *De1* and *De3* ORFs along the TTGHV1 genome. **(B)** Graphical illustration of De2 and its cellular counterpart *Tursiops truncatus* PD-L1. The length of their different domains, the percentage of amino acid identity between them in the two proteins, and the predicted N-glycosylation sites are indicated. **(C)** Alignment of De2 and host PD-L1. The Ig1 and Ig2 domains, stalk (ST), transmembrane region (TM), and cytoplasmic tail (CT) are marked. Signal peptides are not shown. Predicted β-strands in the Ig-like domains are depicted with arrows. Changed amino acids in De2 respect to host PD-L1, potential N-linked glycosylation sites, and contacting residues in the PD-1:PD-L1 heterophilic dimer are indicated. **(D)** Predicted tertiary structure of De2 and host PD-L1 Ig-like domains. β-strands are shown as arrows, and significant structural changes are indicated with red squares.

To search for viral homologs of cellular PD-1 ligands, we employed the human PD-L1 and PD-L2 amino acid sequences to interrogate the viral genome data set available in GenBank (9). We identified De2, a gene product encoded by a γ-herpesvirus that infects bottlenose dolphins (*Tursiops truncatus gammaherpesvirus 1* [TTGHV1]) sharing a high degree (60%) of amino acid identity with human PD-L1. TTGHV1, also known as *Delphinid gammaherpesvirus 1*, is so far the only member of the *Bossavirus* genus, one of the seven genera of the γ-*herpesvirinae* subfamily. The *De2* gene is localized at the left end of the viral genome, between the *De1* and *De3* genes (**Figure 1A**). It is predicted to have a structure similar to that of PD-L1, being a type I membrane protein containing two Ig-like domains (**Figures 1B,C**). The mature viral protein, of 250-amino acids, exhibits an overall identity with the host PD-L1 of 71%, including a remarkable nearly complete identity in its N-terminal Ig-like domain (Ig1; 93%) with the conservation of the 18 residues that are involved in PD-1 interaction (**Figures 1B, C**; 7, 26). Three-dimensional modeling of the structures of De2 and host PD-L1 extracellular Ig-like domains predicted, however, the appearance of a new alpha helix between β-strands C´ and D inside the N-terminal Ig-like domain of De2, due to the presence of Val^74^ and Asp^79^ instead of Leu^74^ and Ser^79^ (**Figures 1C, D**). In addition, significant changes were predicted in the membrane-proximal Ig-like domain (Ig2), in the loops between β-strands A and B, due to substitutions in this region, and between β-strands C and D, being longer in De2 as the result of a 5-aa insertion (**Figures 1C, D**). Finally, an important alteration of De2 regarding host PD-L1 is the loss of the cytoplasmic tail, and consequently the impossibility of transducing survival signals (27). Interestingly, the six N-glycosylation sites are fully conserved in De2 (**Figures 1B, C**).

### De2 is a cell surface protein that efficiently binds PD-1

**Figure 2:**
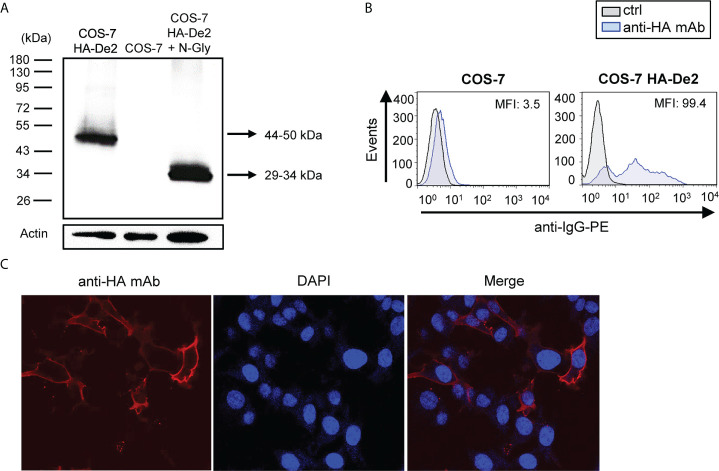
De2 is a highly glycosylated cell surface protein. **(A)** COS-7 cells nontransfected or transfected with HA-De2 were lysed and subjected to SDS-PAGE and Western blot analysis using an anti-HA mAb. When indicated, cell lysates were treated with N-glycosidase F (N-Gly). An anti-β-actin mAb was used as an internal control. Molecular masses in kDa are shown. **(B)** Flow cytometry analysis of COS-7 cells nontransfected or transfected with HA-De2 and stained with an anti-HA mAb or unstained (ctrl), and incubated with an anti-IgG-PE antibody. The MFI is indicated in each histogram. **(C)** COS-7 cells transfected with HA-De2 were fixed and stained with an anti-HA mAb, followed by a biotinylated anti-rabbit IgG and streptavidin-AF555. Nuclei were stained with the DAPI reagent. The cells were examined under a confocal microscope. Single channels and overlaid images are shown. Magnification, x63.

To assess De2 biochemically, the viral gene was cloned with an HA tag at the N-terminus and expressed in COS-7 cells. Analysis of the cell lysates by Western blotting identified a distinct band with a molecular mass of 44-50 kDa, which shifted to a 29-34 kDa band after enzymatic digestion using N-glycosidase-F, supporting its highly glycosylated nature (**Figure 2A**). In addition, flow cytometry and indirect immunofluorescence microscopy data demonstrated the cell-surface localization of De2 (**Figures 2B, C**).

**Figure 3:**
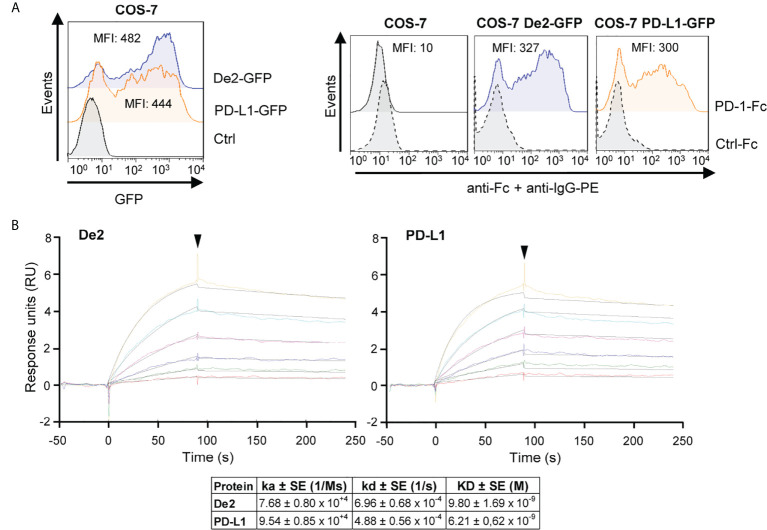
De2 binds host PD-L1. **(A)** COS-7 cells nontransfected (control) or transfected with De2-GFP or PD-L1-GFP were analyzed by flow cytometry to assess the expression of De2 and PD-L1 (left panel), and their interaction with the PD-1-Fc or an unrelated Ctrl-Fc fusion proteins (right panels), using anti-Fc mAb and anti-IgG-PE antibody. The MFI values are indicated in each histogram. **(B)** Representative sensorgrams and fittings obtained for the determination of the kinetic constants of the De2:PD-1 and PD-L1:PD-1 interactions by SPR analysis. Binding and dissociation of several concentrations of analytes (color curves; 0, 12.5, 25, 50, 100, 200 and 400 nM) at 30 μl/min were recorded and adjusted to a 1:1 Langmuir fitting (black lines). The arrowhead points the end of the injection. The mean values ± SEM of the kinetic parameters and derived affinity constants from three independent experiments are indicated in the table below.

To investigate the capacity of De2 to bind PD-1, we expressed host PD-1 as a soluble Ig fusion protein comprising its extracellular domain and the Fc region of human IgG_1_ (PD-1-Fc) and analyzed its interaction with COS-7 cells transfected with De2 tagged with GFP at the C-terminus. Notably, the PD-1 fusion protein efficiently recognized De2-transfected cells, at a similar level as host PD-L1-expressing cells, while no binding to untransfected COS-7 cells could be detected (**Figure 3A**). De2, however, did not bind to human PD-1 (**Supplementary Figure 1**). The binding of De2 and host PD-1 was further assessed by surface plasmon resonance kinetic analyses. To this end, host PD-1-Fc protein was immobilized in a sensor chip and its capacity to bind different concentrations of soluble Ig fusion proteins De2-Fc or control host PD-L1-Fc was examined. The results, shown in **Figure 3B**, evidenced a high affinity and stable interaction between De2 and host PD-1, with an equilibrium dissociation constant (K_D_) of 9.8 nM, in a similar range to that obtained for the interaction of host PD-L1 with PD-1 (K_D_ of 6.2 nM).

### Evolutionary origin of De2

**Figure 4:**
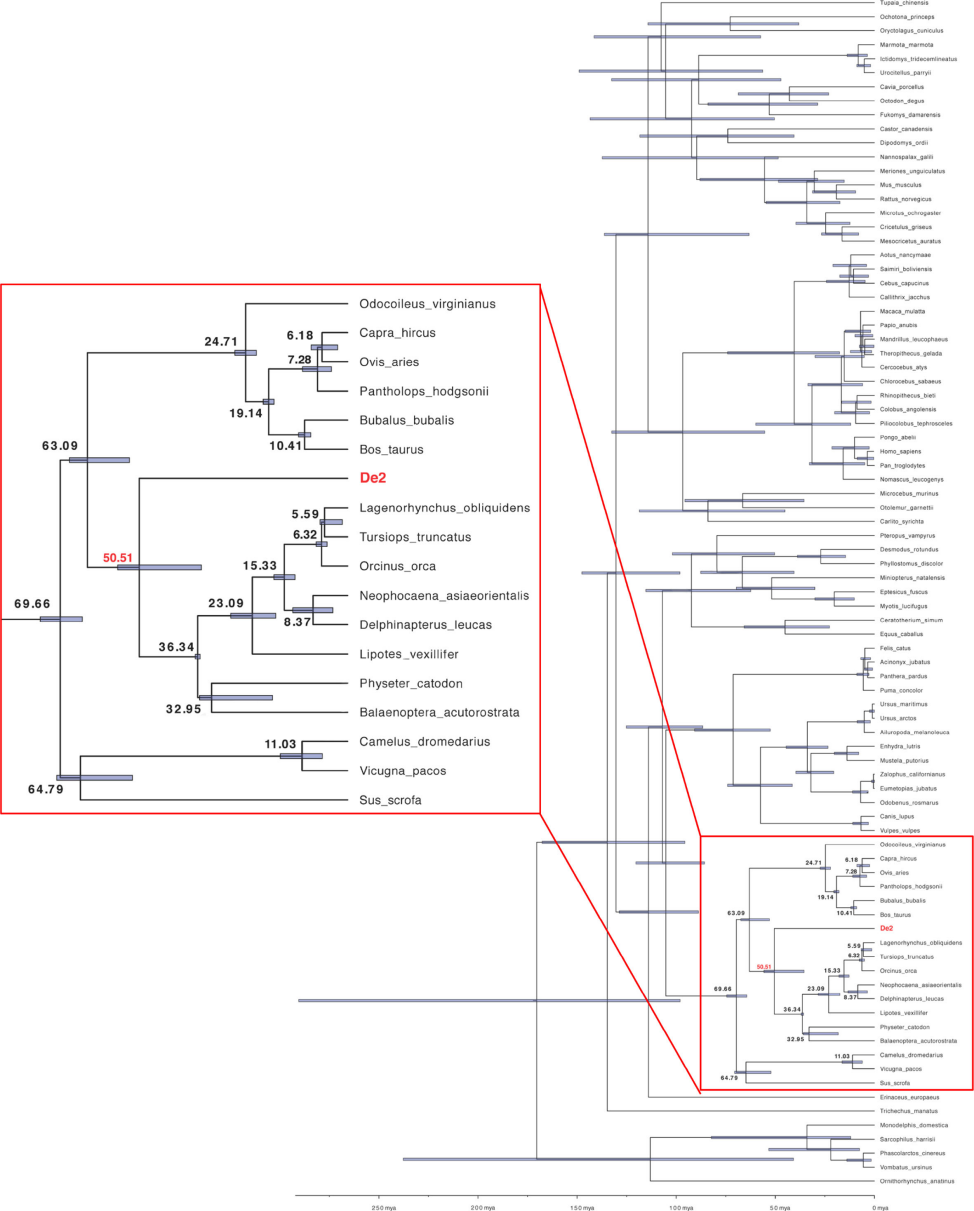
Inferred PD-L1 capturing time, the origin of De2. The PD-L1 capture moment was estimated applying BEAST v1.10.4 with the Bayesian random local clocks method using the cDNA alignment (see Materials and Methods for details). Node numbers (in bold) are the estimated divergence times in million years ago (mya) units. Blue boxed regions depict 95% Highest Posterior Density (HPD) intervals for divergence times. HPD is a Bayesian probability interval similar to a confidence interval. The region marked with a red square is enlarged on the left.

We also estimated the moment of *PD-L1* gene capture that led to *De2* using a Bayesian method with random local clocks implemented in BEAST (13). The results indicated a capture in the cetacean root, after splitting from Pecora, 50 million years ago (**Figure 4**). Thus, the high preservation of the De2 ectodomain (**Supplementary Figure 2**), in particular the N-terminal domain, despite its ancient capture, indicates that strong functional constraints have been operating on this gene.

### De2 attenuates T cell signaling

**Figure 5:**
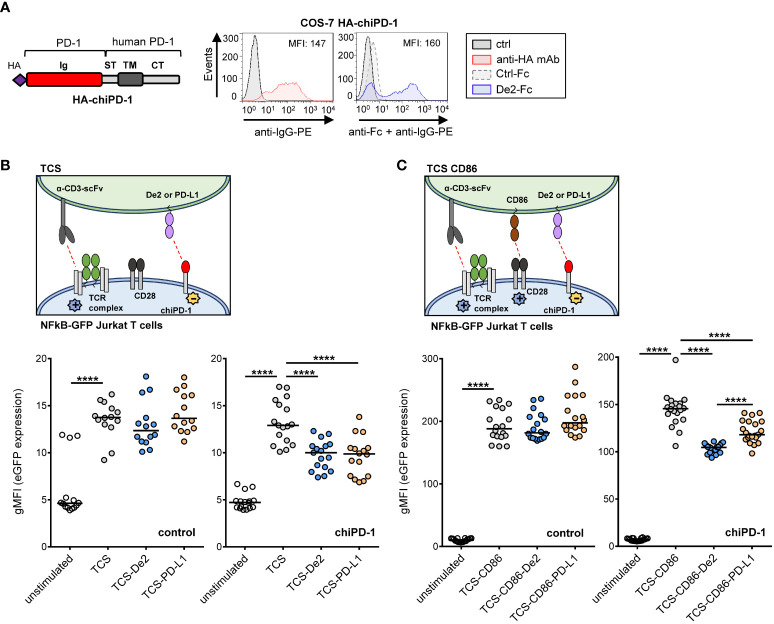
De2 inhibits T cell responses. **(A)** Left, schematic representation of the PD-1 chimera (HA-chiPD-1), composed of the Ig-like domain of host PD-1 and the stalk, transmembrane domain and cytoplasmic tail of the molecule of human PD-1. Right, flow cytometry analysis of the expression of the HA-chiPD-1 protein on COS-7 transfected cells (left panel) using an anti-HA mAb (or left unstained, ctrl) followed by an anti-IgG-PE antibody, and its interaction with De2-Fc or Ctrl-Fc fusion proteins (right panel), employing anti-Fc mAb and anti-IgG-PE antibody. The MFI values are indicated in each histogram. **(B)** Top panel, schematic representation of the assay. Bottom panels, control (left panel) and chiPD-1-expressing (right panel) NFκB::eGFP reporter cells were left unstimulated or stimulated with either TCS or TCS expressing HA-De2 or HA-PD-L1, and eGFP expression was measured by flow cytometry. **(C)** Same as in **(B)**, except that CD86 was also expressed in TCS cells. Reporter activation results shown in **(B, C)** are from six independent experiments performed in triplicates. In **(B, C)**, unpaired t-tests were performed, ****p ≤ 0.0001.

We then hypothesized that surface expression of De2 may impair T-cell mediated responses. To evaluate this, we employed a fluorescence-based cellular approach that we had previously developed to investigate human T-cell co-signaling pathways (24). We first constructed a chimeric HA-tagged PD-1 molecule (chiPD-1), composed of the host PD-1 ectodomain and the transmembrane and cytoplasmic sequences of human PD-1, and confirmed that it was capable to bind De2 in COS transfected cells (**Figure 5A**). We then generated NF-kB::eGFP reporter Jurkat cells stably expressing chiPD-1 (**Supplementary Figure 3A**). In addition, T cell stimulator cells (TCS) co-expressing De2 or host PD-L1 HA-tagged and membrane bound anti-CD3 single-chain fragments (anti-CD3-scFv), able to functionally engage the CD3-TCR complex on the T cell reporters, were engineered (**Supplementary Figure 3B**). Remarkably, in co-culture assays (**Figure 5B**, schematic representation), engagement of chimeric PD-1 by De2 resulted in a significant downregulation of NF-κB activity in the reporter cells, at similar levels than host PD-L1 (**Figure 5B**, bottom right panel). As anticipated, in the control parental reporter cells where chiPD-1 was absent, no effects of De2 or PD-L1 were observed (**Figure 5B**, bottom left panel). Moreover, in co-cultures with TCS-CD86 (**Figure 5C**, schematic representation, and Supplementary **Figure 3C**), which led to a strong enhanced reporter expression *via* the CD28 molecule, De2 also potently suppressed T cell activation through the chimeric PD-1 (**Figure 5C**, bottom right panel). Altogether, these results indicate that De2 functions as a PD-L1 molecule, inducing PD-1 inhibitory signaling.

### Loss of De2 interaction in *cis* with CD80

**Figure 6:**
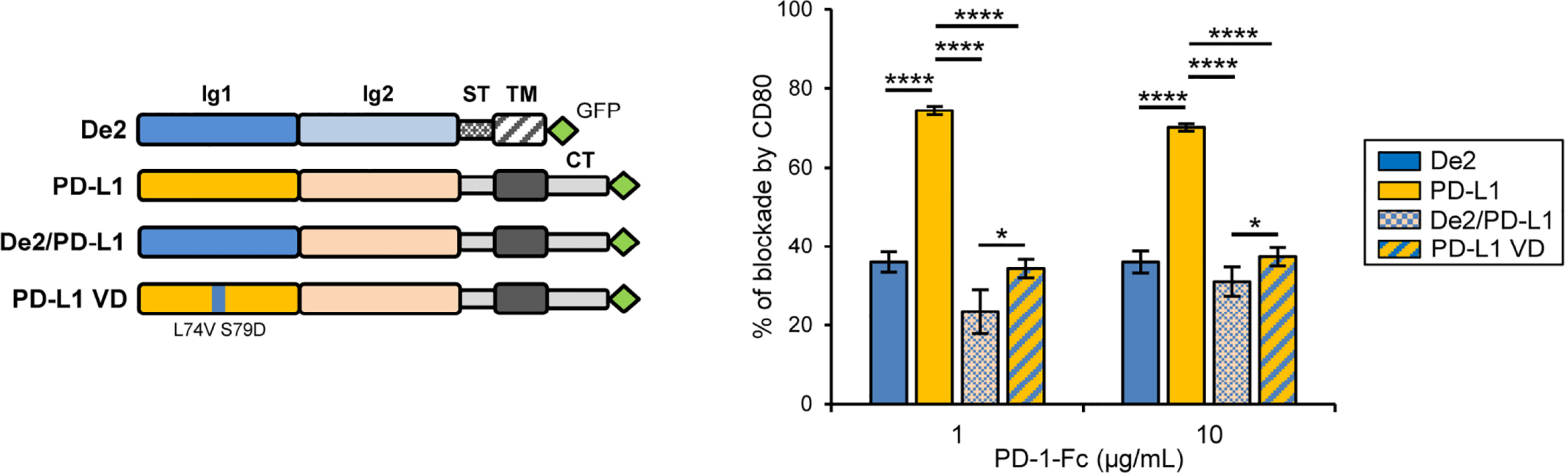
CD80 expression in *cis* minimally alters the interaction of De2 with PD-1 as compared to PD-L1. A schematic representation of De2-GFP, PD-L1-GFP, chimera De2/PD-L1-GFP, and mutant PD-L1 VD-GFP is shown on the left. COS-7 cells co-transfected with HA-CD80 or control empty plasmid, and De2-GFP, PD-L1-GFP, De2/PD-L1-GFP or PD-L1 VD-GFP were incubated with PD-1-Fc protein, and analyzed by flow cytometry. The graph on the right represents the percentages of blockade of PD-1 interactions by CD80. Results shown are in triplicates. Unpaired t-tests were performed, *p ≤ 0.05, ****p ≤ 0.0001.

Finally, it has been reported that on APCs, PD-L1 can interact in *cis* with CD80, preventing PD-1 access to PD-L1 in *trans*, and therefore blocking PD-L1/PD-1 signaling. This *cis*-CD80/PD-L1 interaction restricts PD-1 function during the activation phase of T cells (28, 29). Since a De2 *cis*-interaction of this type may not be beneficial for the immune evasion tactics of the virus, we decided to determine whether CD80 expression affects the binding of the viral protein to PD-1. COS-7 cells were co-transfected with GFP-tagged De2 or PD-L1 along with host CD80 with an N-terminal HA-tag or the HA-control empty vector (HA-ctrl), and incubated with PD-1-Fc. While CD80 was able to strongly interfere with the interaction of PD-1-Fc to PD-L1-GFP, the binding of De2-GFP to PD-1-Fc was largely unaffected by the presence of CD80 (**Figure 6** and **Supplementary Figures 4A–C**). Moreover, when using a chimeric host PD-L1-GFP molecule in which the N-terminal Ig-like domain that participates in CD80 *cis* interaction was swapped by the corresponding domain of De2, the CD80 blocking effects were substantially reduced. Indeed, a similar effect was obtained when mutating host PD-L1 residues L^74^ and S^79^ within the predicted differential loop linking β-strands C’ and D, by the corresponding ones in De2 (PD-L1 VD; **Figure 6** and **Supplementary Figures 4B, C**). Therefore, these results show that while De2 conserves the capacity to efficiently interact with host PD-1, the viral protein has largely lost the “undesired” ability of PD-L1 to interact in *cis* with CD80.

## Discussion

The immune system has evolved sophisticated defense mechanisms to skillfully recognize and control infectious agents such as viruses. As a countermeasure, viruses have been forced to develop specialized means to thwart host immunity and guarantee their survival (1, 30–32). Horizontal gene transfer of host genes into large DNA viruses represents a key driver of viral evolution (32). These captured genes are molded throughout evolution to usually mimic or antagonize the original task, or to accomplish novel immunoregulatory functions (5). In particular, the acquisition and subsequent adaptation of critical components of antiviral defense pathways provides selective advantages to viruses, allowing them to interfere with different elements of host immunity (33). The co-inhibitory molecule PD-1 plays a fundamental role regulating the magnitude and quality of T cell responses. Accordingly, several viruses have been described to upregulate the expression of PD-L1 on hematopoietic cells (34). Here, we report a new viral mechanism to alter the PD-1/PD-L1 axis. We describe the first virus-encoded PD-1 ligand, a PD-L1 molecule refined through viral-host co-evolution, capable to directly bind and functionally engage PD-1.

Our phylogenetic analysis predicted that the host *PD-L1* ancestor that originated *De2* was captured around 50 million years ago. *De2* is intronless, and segments of host PD-L1 introns are not detected inside *De2*. Additionally, there are not traces that could indicate that *De2* neighboring genes in the viral genome were also acquired. These observations suggest that, as most viral homologs, *De*2 was captured by a retrotranscription event (as a cDNA copy) instead of direct recombination between viral and host genomes (3, 35).

We find that De2 is a type-I transmembrane glycoprotein containing an extracellular region that retains the two Ig-domain composition of its cellular counterpart. The N-terminal IgV-like domain, responsible for PD-L1 binding to PD-1, is exceptionally preserved in De2. Accordingly, the viral gene conserves the capacity to efficiently recognize PD-1 and trigger inhibitory signals in T cells. Notably, a subtle alteration in specific residues within a differential loop linking β-strands C´ and D of this domain leads to a substantial decrease in the De2 interaction in *cis* with CD80, an interaction that for PD-L1:CD80 has been reported to limit PD-1 inhibition (28, 29). The function of the Ig-like C2-type domain in PD-L1 is largely unknown, although it has been suggested to exert as a spacer separating the binding site from the cell membrane (7). While this domain has significantly diverged in De2, it has preserved its overall folded structure and size, an aspect that is probably key to maintain the function of the viral protein. A striking feature of De2 is the lack of a cytoplasmic region. There is little information available about the intrinsic signaling functions of PD-L1 apart from engaging PD-1 on T cells (36). However, there is evidence that the cytoplasmic region of PD-L1 can transduce survival signals. Yet, the motifs mediating these effects are not present in the cytoplasmic tail of cetaceans PD-L1 molecules. Therefore, the deletion of the cytoplasmic tail of De2, during viral evolution, may be part of the process of removal of superfluous genetic material, as this region might not present any advantage for viral survival.

The induction of the PD-1/PD-L1 pathway is used by tumor cells to inhibit T cell function and avoid immune destruction. Members of the *γ-herpesvirinae* subfamily, such as Epstein-Barr (EBV) or Kaposi sarcoma-associated herpesvirus (KHSV), are implicated in multiple human malignancies, including a variety of lymphoproliferative and neoplastic disorders. However, nothing is known about the potential role of cetacean γ-herpesvirus encoded proteins in the development of tumors (37, 38). Interestingly, TTGHV1 was isolated from a proliferative cutaneous lesion of a lactating female bottlenose dolphin (39). Thus, it is possible that De2, by preventing the killing of infected cells, contributes to promote both oncogenic progression and viral persistence. Interestingly, EBV and KHSV are among those viruses that have been shown to upregulate the expression of PD-L1 during infection, potentially allowing immune evasion (34, 40, 41).

Our sequence similarity searches did not identify obvious homologs of PD-L1 among other known viruses besides TTGHV1. However, the existence of other cetacean γ-herpesviruses with a *De2* homolog in their genome should be expected. Nevertheless, thus far, the only γ-herpesvirus that infects marine mammals whose genome has been sequenced is TTGHV1. Therefore, genome sequencing and particular gene studies of cetacean herpesviruses will be crucial to identify additional viruses that may bear PD-L1 homologs and to untangle how these viral molecules operate.

Altogether, the discovery of a functional PD-L1 homolog in a γ-herpesvirus reveals a novel viral immunosuppressive strategy and underlines the diversity of mechanisms that viruses have evolved to manipulate crucial immunological pathways. Our findings suggest that PD-L1 homologs may enable viruses to evade adaptive immune responses by promoting T cell dysfunction, favor their replication, and prevent excessive tissue damage.

## Data availability statement

The raw data supporting the conclusions of this article will be made available by the authors, without undue reservation.

## Author contributions

AA and PE conceptualized the study, supervised the work, and interpreted the results. FP, PM-V, and JL performed the experiments and data analysis, and critically discussed the data. DF carried out the phylogenetic analysis and structure modeling. PS contributed to the design and critical interpretation of the results. AA wrote the original draft, and all authors revised the manuscript. All authors contributed to the article and approved the submitted version.

## Funding

This study was supported by the Ministerio de Ciencia e Innovación through the Plan Estatal de Investigación Científica y Técnica y de Innovación 2018-2021 (project reference numbers PID2020-116918RB-100 to AA, and RTI2018-094440-B-100 to PE).

## Acknowledgments

We acknowledge the use of the Advanced Optical Microscopy Facility at the University of Barcelona.

## Conflict of interest

The authors declare that the research was conducted in the absence of any commercial or financial relationships that could be construed as a potential conflict of interest.

## Publisher’s note

All claims expressed in this article are solely those of the authors and do not necessarily represent those of their affiliated organizations, or those of the publisher, the editors and the reviewers. Any product that may be evaluated in this article, or claim that may be made by its manufacturer, is not guaranteed or endorsed by the publisher.
