# Supplementary material for: Discovery of the first PD-1 ligand encoded by a pathogen
Source: Front Immunol. 2022 Sep 13;13:1007334. doi: 10.3389/fimmu.2022.1007334 (PMC9514091; doi:10.3389/fimmu.2022.1007334)

SUPPLEMENTARY INFORMATION

Plasmid constructions. To construct HA-PD-L1, HA-CD80, and HA-De2, first, DNA sequences of the corresponding proteins (from *Tursiops truncatus* or TTGHV1) without their signal peptides were PCR-amplified using as templates chemically synthesized DNAs (Genscript) and primer sets with restriction sites at the 5′ and 3′ ends. The resulting PCR products were inserted into the pGEM-T vector (Promega), subsequently digested, and cloned into the mammalian expression vector pDisplay (Invitrogen). HA-chiPD-1 was constructed by generating two independent PCR products that were subsequently linked by splicing overlap extension (SOE)-PCR. The chemically synthesized DNA of host PD-1 and a human PD-1 expressing plasmid (pCMV6-XL5, OriGene Technologies) were used as templates, and two internal sequence-complementary primers, annealing within the stalks of both molecules, and two external primers were employed for PCR amplification. The resulting SOE-PCR amplified product was inserted into the pGEM-T vector, and subsequently cloned in the pDisplay vector using the restriction sites at 5' and 3' ends of the two external primers. Host PD-1-Fc, De2-Fc and human PD-L1-Fc fusion proteins were obtained by PCR using as templates the chemically synthesized DNAs for PD-1 and De2, and a human PD-L1 plasmid (pCMV6-MYC-DDK, OriGene Technologies), respectively, and specific primer sets with restriction sites at the 5′ and 3′ ends. The PCR-amplified products were inserted into pGEM-T, and finally cloned into pCI-neo Fc vector, as described before (17). De2-GFP and PD-L1-GFP were generated by PCR employing as templates the chemically synthesized DNAs and primer sets with restriction sites at the 5′ and 3′ ends. The resulting PCR products were introduced into the pGEM-T vector, subsequently digested, and inserted in frame with the GFP at the C-terminal end into the pEGFP-N3 plasmid (BD Biosciences Clontech). De2/PD-L1-GFP chimera was constructed as follows: first, independent PCR fragments of De2 or PD-L1 were generated using as templates De2-GFP or PD-L1-GFP, two external primers, at the N-terminal of De2 or the C-terminal of PD-L1, and two internal complementary primers annealing at the end of De2 Ig1 and the beginning of PD-L1 Ig2 domains. SOE-PCR was then performed to join the two PCR fragments obtained for each template using the external primers. These new PCR products were inserted into the pGEM-T vector, and cloned in frame with the GFP at the C-terminal end into the pEGFP-N3 plasmid. PD-L1 VD-GFP was constructed by generating two independent PCR products, using PD-L1 GFP as template, two external primers at the N- and C-terminal of PD-L1, and two internal complementary primers containing the indicated point mutations. SOE-PCR was performed to anneal the two PCR products using the external PD-L1 primers. The product was introduced into the pGEM-T vector, and finally cloned in frame into the pEGFP-N3 plasmid. All PCR reactions were performed under the following conditions: 1 cycle at 94°C for 5 min; 30 cycles of 1 min at 94°C, 1 min at 51°C, and 1 min at 72°C; and 1 cycle at 72°C for 10 min. For the annealing reactions, the conditions were: 6 cycles of 5 min at 94 ºC; 1 cycle at 51 ºC for 1 min; 1 cycle at 72 ºC for 1 min; and 1 cycle of 10 min at 72 ºC. The identification of all recombinant plasmids was confirmed by DNA sequencing, and when required, the correct orientations of the cloned inserts were assessed employing different restriction sites. The transcript annotations for each chemically synthesized DNA were those of GenBank: YP_009388506.1 for De2, XP_004313647.1 and XP_033711703.1 for *Tursiops truncatus* PD-L1 and CD80, respectively, and XP_026971743.1 for *Lagenorhynchus obliquidens* PD-1. The expression constructs for the reporter and T cell stimulator cells were generated as follows: chiPD-1 and De2 were PCR amplified from the pDisplay constructs (including their HA tags) using specific primers and then cloned into the lentivirus vector pHR and the retroviral vector pCJK2,

respectively. PD-L1 was gene synthesized with an N-terminal HA tag (TWIST biosciences) and cloned into pCJK2. The primers used for the generation of all plasmids are included in Supplementary Table 1.

SUPPLEMENTARY FIGURE LEGENDS

Supplementary Figure 1. De2 does not interact with human PD-1. Flow cytometry analysis of COS-7 cells transfected with human PD-1, unstained (grey histogram) or stained with an anti-PD-1 mAb (red histogram), followed by anti-IgG-PE to determine human PD-1 expression, or incubated with De2-Fc (blue histogram), human PD-L1-Fc (green histogram), or an unrelated control Fc (Ctrl, dashed empty histogram) fusion protein, followed by anti-Fc and anti-IgG-PE mAbs to assess the interaction between PD-1 and the Fc proteins. The MFI values are indicated in each histogram.

Supplementary Figure 2. Alignments of De2 and cetacean PD-L1 homologs. Protein sequence alignments of De2, and PD-L1 from *Tursiops truncatus*, *Lagenorhynchus obliquidens*, *Orcinus orca*, *Neophocaena asiaeorientalis*, *Delphinapterus leucas*, *Lipotes vexillifer*, *Physeter catodon*, and *Balaenoptera acutorostrata*.

Supplementary Figure 3. Expression of chiPD-1 in reporter cells and De2 and PD-L1 in TCS and TCS-CD86 cells. (A) Flow cytometry analysis of control or HA-chiPD-1-expressing NFκB::GFP Jurkat reporter cells, using the anti-HA mAb followed by an anti-IgG-APC antibody. (B, C) Flow cytometry analysis of CD3-scFv-expressing TCS (B) or TCS CD86 (C) nontransduced (control) or transduced with HA-tagged De2 or PD-L1, and unstained or stained with an anti-HA mAb followed by an anti-IgG-APC or an anti-CD14-APC mAb (for anti-CD3-scFv detection). The MFI values are indicated in each histogram.

Supplementary Figure 4. Interaction of host PD-1 with De2 or PD-L1 in the presence of host CD80. (A) Example of the gating strategy used in the flow cytometry analysis. Data shown correspond to the analysis of COS-7 cells transfected with De2-GFP as indicated in (B). (B) COS-7 cells were transfected with De2-GFP, PD-L1-GFP, De2/PD-L1-GFP, or PD-L1 VD-GFP, and HA-CD80 or control HA-empty plasmid (HA-ctrl), and the expression of GFP (left panel) or HA using an anti-HA mAb followed by an anti-IgG-PE antibody (right panel) was analyzed by flow cytometry. (C) COS-7 cells transfected as indicated in (B) were stained with PD-1-Fc fusion protein and analyzed by flow cytometry (selected cells as indicated in B, left panel). Unstained transfected cells (ctrl) show background staining. The MFI values are indicated in each histogram.

Name	Length	Sequence	Use
BglIIHA-De2For	27	5’ AGATCTTTCACTGTCACGGTCCCCAAG 3’	HA-De2
SalIHA-De2Rev	27	5’ GTCGACTTATTTCACATAAAAGATGAG 3’	
BglIIHA-ttPDL1For	27	5’ AGATCTTTTACTATCACAGTTCCCAAG 3’	HA-PD-L1
SalIHA-ttPDL1Rev	27	5’ GTCGACTTACGTCTCCTCAAATTGTGT 3’	
BglIIHA-ttCD80For	27	5’ AGATCTATTATCCAGGTGACCAAAACA 3’	HA-CD80
SalIHA-ttCD80Rev	27	5’ GTCGACCTATGTAGATGGGGAAATCCT 3’	
BglIIHA-loPD1For	27	5’ AGATCTCTAGATGCCCCCAGCAGGCCC 3’	HA-chiPD-1
SalIHA-chiPD1Rev	27	5’ GTCGACTCAGAGGGGCCAAGAGCAGTG 3’	
SOEchiPD1Rev	30	5’ CACCAGGGTTTGGAACTGGCCTTCGGTCCT 3’	
SOEchiPD1For	30	5’ AGGACCGAAGGCCAGTTCCAAACCCTGGTG 3’	
loPD1FcFor	28	5’ GGATCCACTAGATGCCCCCAGCAGGCCC 3’	PD-1-Fc
loPD1FcRev	37	5’ GGATCCACTTACCTGTCTGTAACTGGCCTTCGGTCCT 3’	
De2FcFor	28	5’ GGATCCATTCACTGTCACGGTCCCCAAG 3’	De2-Fc
De2FcRev	37	5’ GGATCCACTTACCTGTGGACCGCGGGTACGTCCAGCG 3’	
hPDL1FcFor	28	5’ AGATCTCTTTACTGTCACGGTTCCCAAG 3’	human PD-L1-Fc
hPDL1FcRev	37	5’ AGATCTACTTACCTGTCCTTTCATTTGGAGGATGTGC 3’	
NheIDe2For	27	5’GCTAGCATGTTGCTGTGTTTTTTAGCT 3’	De2-GFP, De2/PD-L1-GFP
BamHIDe2noCSRev	27	5’ GCATCCTTTCACATAAAAGATGAGTAT 3’	De2-GFP
NheIttPDL1For	27	5’ GCTAGCATGAGGATATACAGTATCTTT 3’	PD-L1-GFP
BglIIttPDL1noCSRev	30	5’ AGATCTCGTCTCCTCAAATTGTGTATCATT 3’	PD-L1-GFP, De2/PD-L1-GFP
SOEDe2/ttPDL1For	30	5’ TACCGCAAAATCAACCAAACAATTTCTGTG 3’	De2/PD-L1-GFP
SOEDe2/ttPDL1Rev	30	5’ CGGTACGACGCGTTAACCTTTAACGTGATC 3’	
SOEttPDL1L74VS79DFor	33	5’ GTGAATGTTCAACACGACAGCTACAACCAGAGG 3’	PD-L1 VD-GFP
SOEttPDL1L74VS79DRev	33	5’ GTCGTGTTGAACATTCACGTCTTCCTCCCCATT 3’	
Xho_pDisplay_for	37	5´GGCGGGGCTCGAGACCATGGAGACAGACACACTCCTG 3´	HA-chi-PD1 pHR
BamHI_chiPD1_rev	33	5´ GCGGGGGGATCCGAGGGGCCAAGAGCAGTGTCC 3´	
HindIII_pDisplay_for	36	5´GCGCCCAAGCTTACCATGGAGACAGACACACTCCTG 3´	HA-De2 pCJK2
NotI_De2_rev	44	5´GCGCCCGCGGCCGCTTATTTCACATAAAAGATGAGTATTACGAC 3´	
SfiI_PDL1_for	22	5´GCGCCCGGCCATTACGGCCATG 3´	HA-PD-L1 pCJK2
Sfi_PDL1_rev	24	5´GCGCCCGGCCGAGGCGGCCTTACG 3´	

Supplementary Table 1. Primer sets employed in the study

Supplementary Figure 1

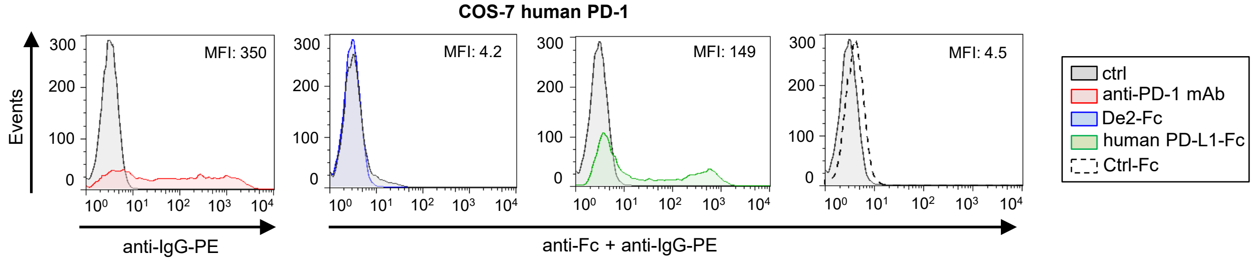

Supplementary Figure 2

```
De2                           M----LLCFLALVAFWPCLLPAFTVTVPKDLYVVEYGGNVTLECRFPVDKQLNLLALVVY
Tursiops_truncatus            MRIYSIFTFMA----YSCLLKAFTITVPKDLYVVEYGSNVTLECRFPVDKQLNLLALVVY
Lagenorhynchus_obliquidens    MRIYSIFTFMA----YCCLLKAFTITVPKDLYVVEYGSNVTLECRFPVDKQLNLLALVVY
Orcinus_orca                  MRIYSIFTFMA----YCCLLKAFTITVPKDLYVVEYGSNVTLECRFPVDKQLNLLALVVY
Neophocaena_asiaeorientalis   MRIYSIFTFMA----YCCLLKAFTITVPKDLYVVEYGSNVTLECRFPVDKQLNLLALVVY
Delphinapterus_leucas         MRIYSIFTFMA----YCCLLKAFTITVPKDLYVVEYGNNVTLECRFPVDKQLNLLALVVY
Lipotes_vexillifer            MRIYSIFTFMA----YCCLLKAFTITVPKDLYVVEYGSNVTLECRFPVDKQLNLLALVVY
Physeter_catodon              MRIYSIFTFMA----YCCLLKAFTVTVPKDLYVVEYGSNVTLECIFPVDKQLNLLALVVY
Balaenoptera_acutorostrata    MRIYSIFTFMA----YCCLLKAFTITVPKDLYVVEYGSNVTLECKFPVDKQLNLLALVVY
                              *    :: *:*    : *** ***:************.****** ***************

De2                           WEMEDKKIIQFVNGEEDVNVQHDSYKNRVTLLKNQLPLGKAALQITDVKLQDAGIYCCLI
Tursiops_truncatus            WEMEDKKIIQFVNGEEDLNVQHSSYNQRALLLKNQLPLGKAALQITDVKLQDAGIYCCLI
Lagenorhynchus_obliquidens    WEMEDKKIIQFVNGEEDLNVQHSSYNQRALLLKNQLPLGKAALQITDVKLQDAGIYCCLI
Orcinus_orca                  WEMEDKKIIQFVNGEEDLNVQHSSYNQRALLLKNQLPLGKAALQITDVKLQDAGIYCCLI
Neophocaena_asiaeorientalis   WEMEDKKIIQFVNGEEDLNVQHSSYNQRALLLKNQLPLGKAALQITDVKLQDAGIYCCLI
Delphinapterus_leucas         WEMEDKKIIQFVNGEEDLNVQHSSYNQRALLLKNQLPLGKAALQITDVKLQDAGIYCCLI
Lipotes_vexillifer            WEMEDKKIIQFVNGEEDLNVQHSSYNQRALLLKDQLSLGKAALQITDVKLQDAGIYCCLI
Physeter_catodon              WEMEDKKIIQFVNGEEDLNVQHSSYNQRALLLKDQLSLGKAALQITDVKLQDAGIYCCLI
Balaenoptera_acutorostrata    WEMEDKKIIQFVNGEEDLNVQHSSYNQRALLLKDQLSLGKAALQITDVKLQDAGIYCCLI
                              *****************:****.**::*. ***:**.***********************

De2                           SYGGADYKRITLKVNASYRKINGSVAAVPGSSGHELTCQSEGYPEAEVIWTRGNASGKGG
Tursiops_truncatus            SYGGADYKRITLKVNASYRKINQTISVDPVTSEHELMCQAEGYPEAEVIWTSSDHR----
Lagenorhynchus_obliquidens    SYGGADYKRITLKVNASYRKINQTISVDPVTSEHELMCQAEGYPEAEVIWTNSDHR----
Orcinus_orca                  SYGGADYKRITLKVNASYRKINQTISVDPVTSEHELMCQAEGYPEAEVIWTSSDHR----
Neophocaena_asiaeorientalis   SYGGADYKRITLKVNASYRKINQTISVDPVTSEHELMCQAEGYPEAEVIWTSSDHR----
Delphinapterus_leucas         SYGGADYKRITLKVNASYRKINQTISVDPVTSEHELMCQAEGYPEAEVIWTSSDHR----
Lipotes_vexillifer            SYGGADYKRITLKVNASYRKINQTISVDPVTSEHELMCQAEGYPEAEVIWTSSDHR----
Physeter_catodon              SYGGADYKRITVKVNAPYRKINQTIYVDPVTSEHELTCQAEGYPEAEVIWTSSDHR----
Balaenoptera_acutorostrata    SYGGADYKRITLKVNAPYRKINQTISVDPVTSEHELTCQAEGYPEAEVIWTSSDHR----
                              ***********:****.***** :: . * :* *** **:*********** .:

De2                           PALSGKTTVTSSKREEKLFNVTSVLRINTTANEIFYCIFRRLGHEENSTAELVIPGPDWA
Tursiops_truncatus            -VLSGKTTITSSKREEKLFNVTSTLRINTTANEIFYCIFRRLGHEENSTAELVIPEPYPD
Lagenorhynchus_obliquidens    -VLSGKTTITSSKREEKLFNVTSTLRINTTANEIFYCIFRRLGHEENSTAELVIPEPYPD
Orcinus_orca                  -VLSGKTTITSSKREEKLFNVTSTLRINTTANEIFYCIFRRLGHEENSTAELVIPEPYPD
Neophocaena_asiaeorientalis   -VLSGKTTITSSKREEKLFNVTSTLRINTTANEIFYCIFRRLGHEENSTAELVIPEPYPD
Delphinapterus_leucas         -VLSGKTTITSSKREEKLFNVTSTLRINTTANEIFYCIFRRLGHEENSTAELVIPEPYPD
Lipotes_vexillifer            -VLSGKTTITSSKREEKLFNVTSTLRINTTANEIFYCIFRRLGHEENSTAELVIPEPYPD
Physeter_catodon              -VLNGKTTITSSKREEKLFNVTSTLRINTTANEIFYCIFRRLGHEENSTAELVIPEPYPD
Balaenoptera_acutorostrata    -VLSGKTTITSSRREEKLFNVTSTLRINTTANEIFYCIFRRLGHEENSTAELVIPEPYPD
                               .*.****:***:**********.******************************* *

De2                           PGRWTYPRSRLGVPGAFLAVLVIVVILIFYVK------------------------------
Tursiops_truncatus            PAK---KRTHLVILGALLLFLSVTLTIIFYLKRDVEVMDMEKCGTRDMNSKQQNDTQFEET-
Lagenorhynchus_obliquidens    PAK---KRTHLVILGALLLFLSVTLTIIFYLKRDVEVMDMEKCGTRDMNSKQQNDTQFEET-
Orcinus_orca                  PAK---KRTHLVILGALLLFLSVTLTIIFYLKRDVEVMDMEKCGTRDMNSKQQNDTQFEET-
Neophocaena_asiaeorientalis   PAK---KRTHLMILGALLLFLSVTLTIIFCLKRDVGVMDMEKCGTRDMNSKQQNDTQFEET-
Delphinapterus_leucas         PAK---KRTHFVILGALLLFLSVTLTIIFCLKR---------CGSDGYGKMWHPRYELKAAK
Lipotes_vexillifer            PAK---KRTHLVILGALLLFLSVTLTIIFCLKRDVGVMDMGKCGTRDMSSKQQNDTQFEET-
Physeter_catodon              PAK---KRTHLVILGALLLFLSVTLTIIFCLKRDVRVMDVEKCGTQDMNSKQQNDTQFEET-
Balaenoptera_acutorostrata    PAK---KRTHLVILGALLLFLSVTLTIIFCLKRDVRMMDVEKCGTRDMNSKQQNDTQFEET-
                              *.:    *::: : **:* .* :.: :** :*
```

Supplementary Figure 3

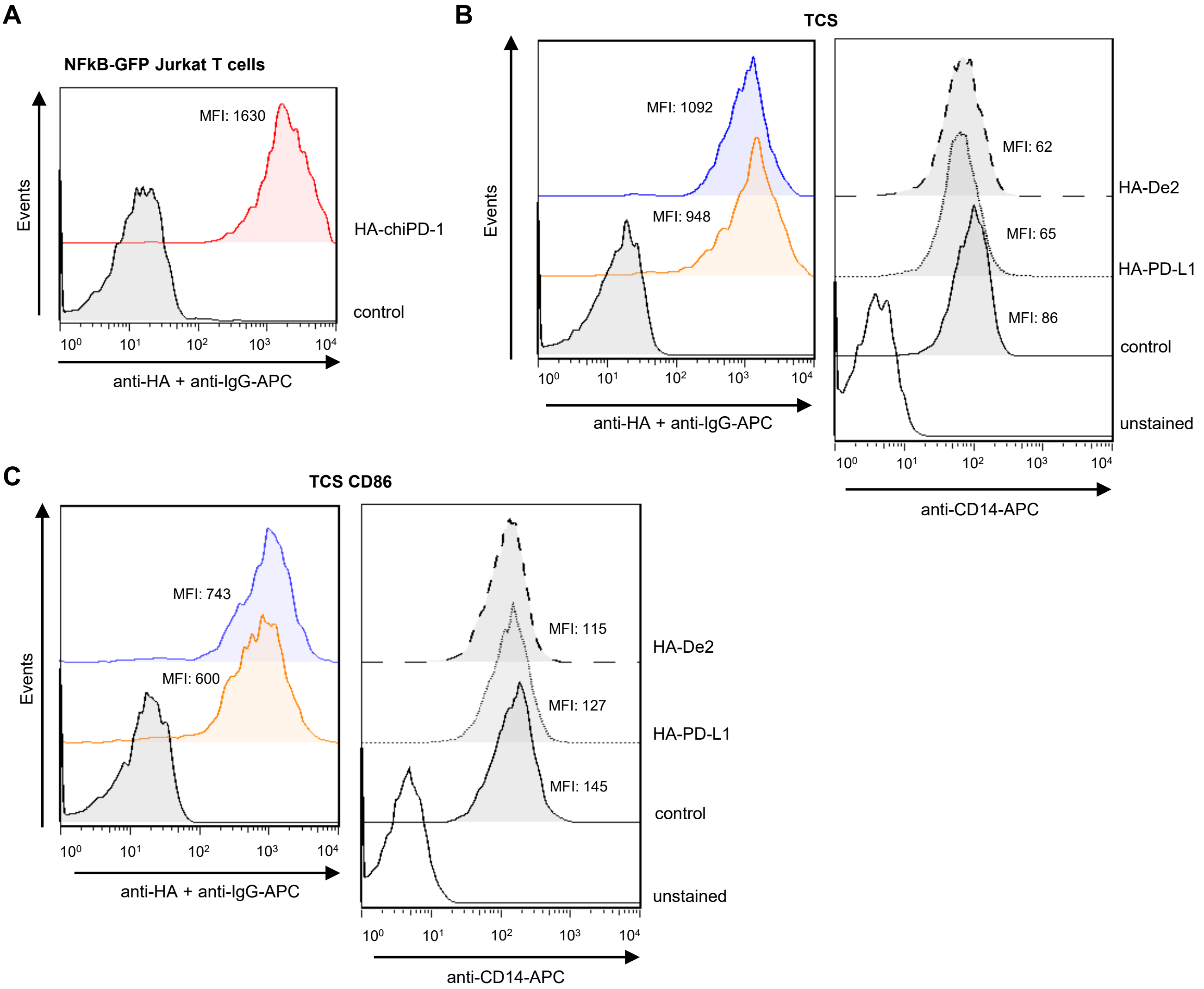

Supplementary Figure 4

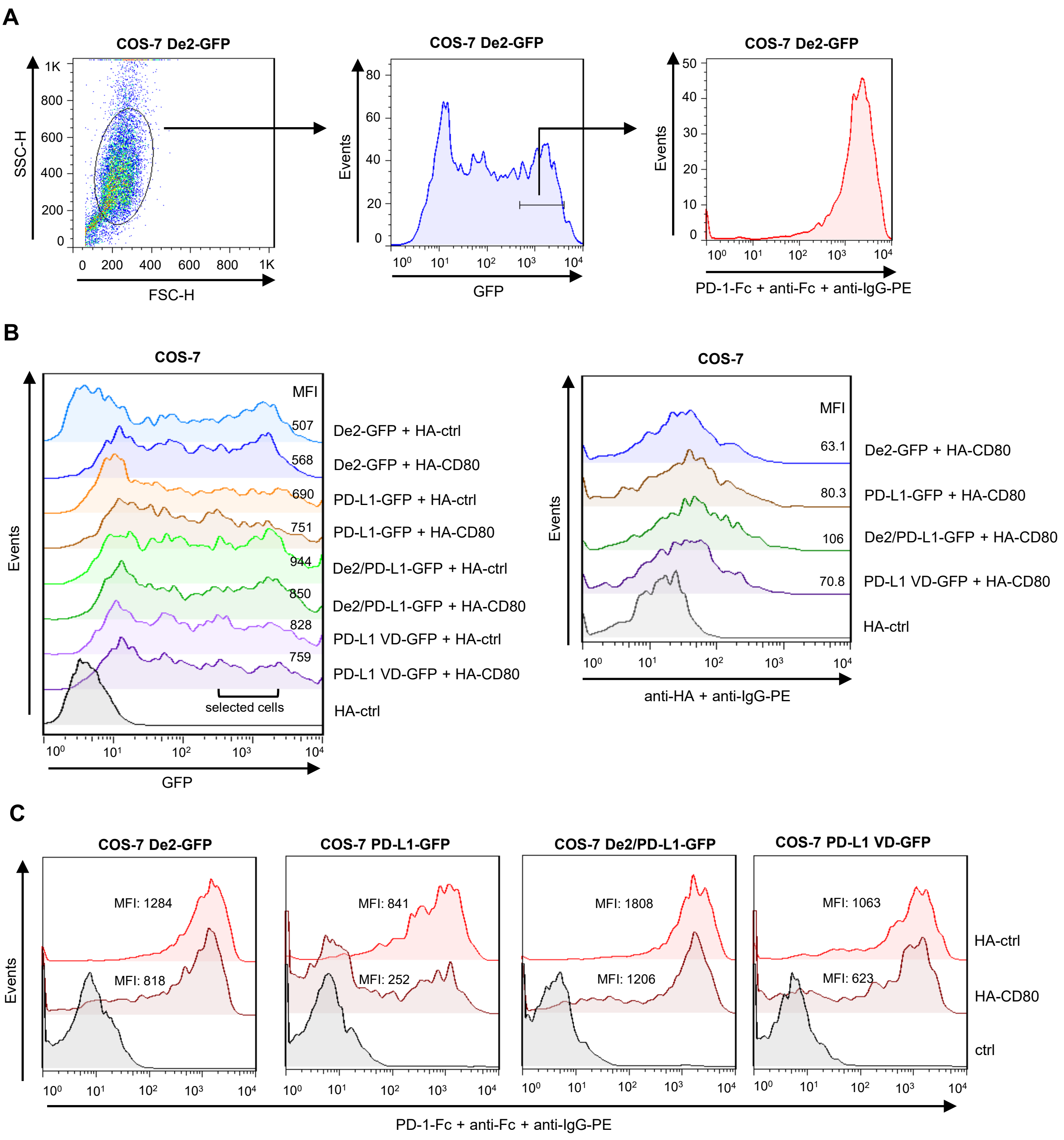

Supplement: Supplementary file 1 [file DataSheet_1.pdf]
